# Differential gene expression analysis and physiological response characteristics of passion fruit (*Passiflora edulis*) buds under high-temperature stress

**DOI:** 10.7717/peerj.14839

**Published:** 2023-02-02

**Authors:** Hongli Wang, Jiucheng Zhao, Miao Lai, Yingqing Zhang, Wenwu Qiu, Yanyan Li, Hailian Tu, Qichang Ling, Xinfeng Fu

**Affiliations:** 1 Qinzhou Branch of Guangxi Academy of Agricultural Sciences/Qinzhou Institute of Agricultural Sciences, Qinzhou, China; 2 Institute of Horticulture, Guangxi Academy of Agricultural Sciences, Nanning, China

**Keywords:** Passion fruit, High-temperature stress, Chlorophyll fluorescence, RNA-seq, Differential expression gene, Pathway

## Abstract

High temperature in summer is an unfavorable factor for passion fruit (*Passiflora edulis*), which can lead to restricted growth, short flowering period, few flower buds, low fruit setting rate, severe fruit drop, and more deformed fruit. To explore the molecular physiology mechanism of passion fruit responding to high-temperature stress, we use ‘Zhuangxiang Mibao’, a hybrid passion fruit cultivar, as the test material. Several physiological indicators were measured and compared between high-temperature (average temperature 38 °C) and normal temperature (average temperature 25 °C) conditions, including photosynthesis, chlorophyll fluorescence parameters, peroxidase activity (POD), superoxide dismutase activity (SOD) and malondialdehyde content. We performed RNA-seq analysis combined with biochemistry experiment to investigate the gene and molecular pathways that respond to high-temperature stress. The results showed that some physiological indicators in the high-temperature group, including the net photosynthetic rate, stomatal conductance, intercellular CO_2_ concentration, transpiration rate, and the maximum chemical quantum yield of photosystemII (PSII), were significantly lower than those of the control group. Malondialdehyde content was substantially higher than the control group, while superoxide dismutase and superoxide dismutase activities decreased to different degrees. Transcriptome sequencing analysis showed that 140 genes were up-regulated and 75 genes were down-regulated under high-temperature stress. Gene Ontology (GO) and Kyoto Encyclopedia of Genes and Genomes (KEGG) annotation analysis of differentially expressed genes revealed many metabolic pathways related to high-temperature stress. Further investigation revealed that 30 genes might be related to high-temperature stress, such as *chlorophyllide a oxygenase (CAO)*, *glutathione* (*GSH*), *WRKY transcription factors* (*WRKY*), and *heat shock protein* (*HSP*), which have also been reported in other species. The results of real-time fluorescence quantitative PCR and RNA-seq of randomly selected ten genes are consistent, which suggests that the transcriptome sequencing results were reliable. Our study provides a theoretical basis for the mechanism of passion fruit response to high-temperature stress. Also, it gives a theoretical basis for the subsequent breeding of new heat-resistant passion fruit varieties.

## Introduction

Passion fruit is a perennial evergreen vine-like fruit tree, considered a tropical high-quality fruit tree with excellent development prospects based on its high nutritional, ornamental, and medicinal value (Wang et al., 2022). At the same time, passion fruit is also a typical economic crop that is easy to grow and difficult to manage. One of the main reasons it is difficult to manage is that heat damage caused by environmental changes often occurs and causes devastating damage to the industry. The optimal growth temperature of passion fruit is 20–30 °C. Above 30 °C, photosynthesis will decrease. If the temperature exceeds 35 °C, a series of physiological lesions will appear in the plant, such as abnormal flower organs, leaves, fruit tissue, deformity, necrosis, and other sunburn symptoms. In addition, the fruit setting rate drops to 10% to 15% of when it is grown at normal temperature (Qin et al., 2017; Zheng & Wei, 2018; Lan, Shi & Li, 2021). High temperatures not only change the phenotypic traits of the fruit but also cause the imbalance of cell homeostasis in the plant body and inhibit growth and development. High temperatures also lead to large-scale dead seedlings, seriously affecting their quality and yield and causing enormous economic losses.

With the intensification of the greenhouse effect, the high-temperature stress caused by global warming has become an increasingly severe challenge for the modern agricultural production system. It is of great scientific significance and practical urgency to study the mechanism of heat regulation in plants. The research on plant heat resistance has become an important field in agricultural scientific research in China and elsewhere (Chen & Guo, 2013; Akhoundnejad, Dasgan & Karabiyik, 2020; Ma et al., 2021; Ren et al., 2021). However, research on the response of passion fruit to high-temperature heat damage is more recent. Studies revealed the effect of high temperature on yield, quality, growth, and development, and the effects of physiological and biochemical characteristics (Tian et al., 2020; Tian et al., 2021; Huang et al., 2021); however the molecular mechanisms of passion fruit response to high-temperature stress are still unknown.

The emergence and development of RNA-seq technology have greatly accelerated plant science research at the molecular level and provided new methods and ideas for a better understanding of the mechanism of heat resistance regulation in passion fruit. This technology is increasingly used to construct transcriptional maps under plant stress and to screen stress-responsive plant genes. Therefore, it is possible to use RNA-seq technology to explore the heat-resistant response mechanism of passion fruit under high-temperature stress. This technology has revealed the high-temperature stress response mechanism of many plants. Huang et al. (2022) performed a transcriptome sequencing analysis of *Vitis vinifera* under high-temperature stress and screened *BI1* with differential gene expression induced by high-temperature stress. By constructing a fusion expression vector to analyze the subcellular localization of *BI1* and using a yeast two-hybrid system and other technical analyses, they found that *BI1* was a positive regulator of the *Vitis vinifera* response to high-temperature stress (Huang et al., 2022). A previous study by Chen, Han & Ren (2021) showed that many *heat shock protein (HSP)* family genes are highly up-regulated when *Cucumis sativus* is subjected to high-temperature stress, and *AP2*, *MYB*, *WRKY*, *bHLH*, and *HSF* transcription factors show different expression patterns under high-temperature stress. In addition to conventional crops, the heat tolerance mechanisms of medicinal plants such as *Pinellia ternata*, *Mentha canadensis*, and *Artemisia annua* have also been revealed by transcriptome sequencing (Lu et al., 2018; Zhu, Li & Gao, 2020; Guo et al., 2022).

Therefore, to investigate the molecular regulation mechanism of passion fruit in response to high-temperature stress, we selected the high-temperature-sensitive passion fruit variety ‘Zhuangxiang Mibao’ cultivated locally in the Guangxi Zhuang Autonomous Region, as the test material. By analyzing the physiological characteristic under high-temperature and normal temperature conditions combined with transcriptome sequencing technology, the essential functional genes and regulatory factors involved in the high-temperature response of passion fruit were identified. Our study provides essential theoretical and practical significance for improving the tolerance to high temperatures and for cultivating new varieties of passion fruit with high-temperature resistance through a genetic approach.

## Materials and Methods

### Plant material

**Figure 1:**
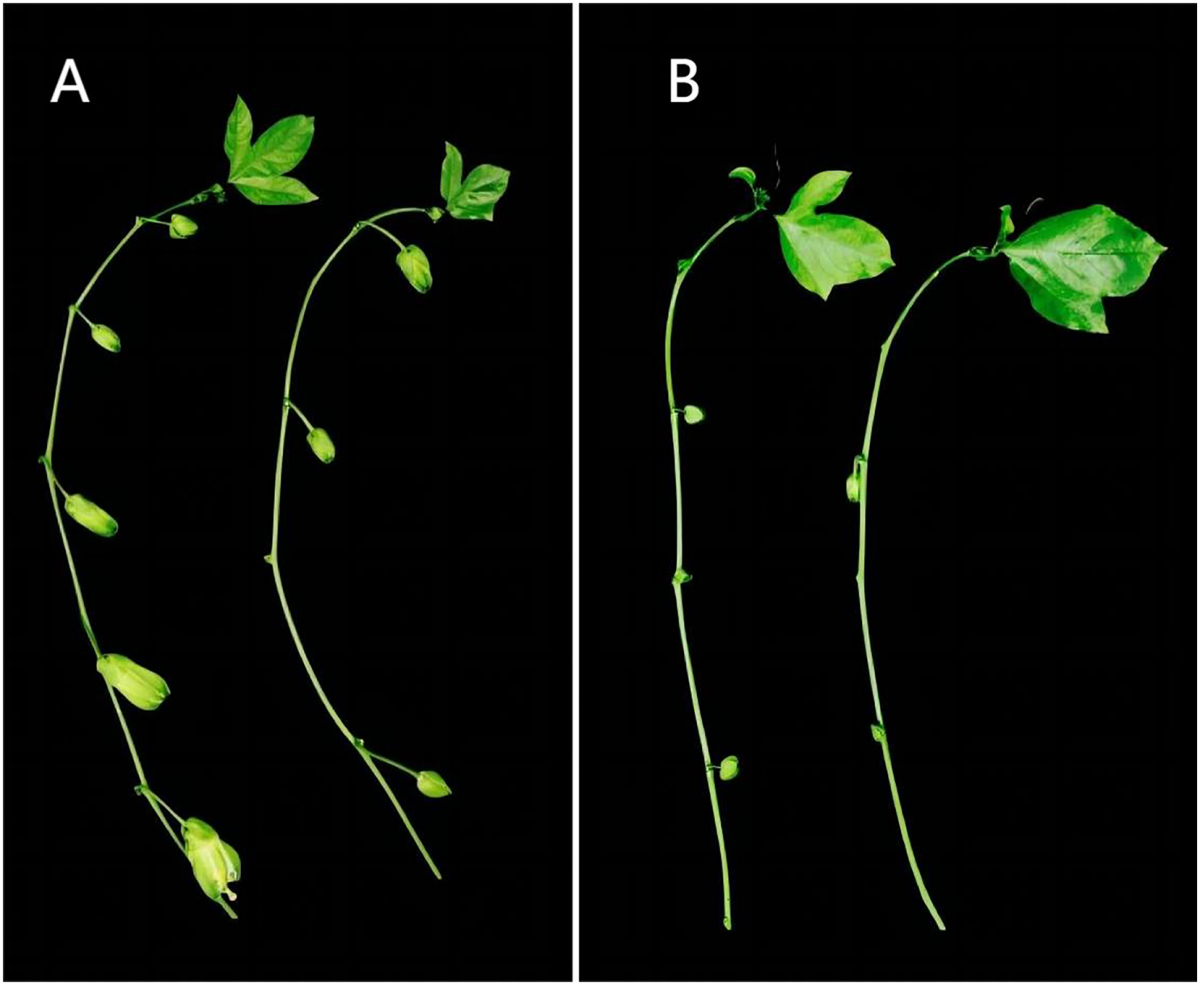
Differences of flower bud growth between secondary and tertiary vines of passion fruit in different environments in the same period. (A) Number of secondary vine buds; (B) number of tertiary vine buds. Left, CK group; right, HT group. Under the two experimental conditions, there are significant differences in the number and growth of flower buds between the second-class vine and the third-class vine. The CK group has more flower buds and larger buds, while the HT group has less flower buds and smaller buds.

The test material ‘Zhuangxiang Mibao’ was planted in the Jiulong Experimental Base of the Agricultural Science Research Institute, Qinzhou, Guangxi, in February 2022. Two hundred grafted seedlings were grown in the open air or experimental greenhouse, where the average daily temperature in May was 25 °C and 38 °C, respectively. Under the two experimental conditions, there were significant differences in the number of flower buds between secondary and tertiary cranium (Fig. 1). The young flower buds of ‘Zhuangxiang Mibao’ with the same experimental field, growth potential, external morphological characteristics, and seedling height of about 150 cm were selected. The surfaces of the selected young flower buds were rinsed with 75% ethanol, dried with absorbent paper, and aliquoted with 100 mg per capped storage tube. The capped storage tube was quickly frozen in liquid nitrogen and stored at −80 °C. Three biological replicates were set and numbered for six samples to filter false positive signals. Under the HT condition, the three biological replicates were HT_1_, HT_2_, and HT_3_; Under the CK condition, the three biological replicates were CK_1_, CK_2_, and CK_3_.

### Determination of physiological index

To ensure the accuracy of photosynthetic index determination, the leaves with the same position, moisture, and nutritional status and without insect spots and disease spots were selected, and the experiments were performed at 9:00–11:30 in the morning when the photosynthesis induction period reached a steady state. A portable photosynthesis instrument (LI-6400XT) was used in the experimental base to determine the net photosynthetic rate (Pn), stomatal conductance (Gs), concentration of intercellular CO_2_ (Ci), transpiration rate (Tr), and other photosynthetic indicators. After changing the fluorescent leaf chamber probe, the chlorophyll fluorescence parameter values were measured, including dark-adapted maximum fluorescence yield (Fm), dark-adapted minimum fluorescence yield (Fo), light-responsive maximum fluorescence yield (Fm′), the minimum fluorescence yield of photoreaction (Fo′), and the steady-state fluorescence yield (Fs). The photochemical potential activity of photosystemII (PSII) (Fv/Fo) and the maximum photochemical quantum efficiency of PSII (Fv/Fm) were calculated based on the chlorophyll fluorescence parameters. Peroxidase (POD) activity, superoxide dismutase (SOD) activity, and malondialdehyde (MDA) content were detected indoors using biochemical kits (Solarbio, Beijing, China). Measured data were analyzed using DPS v1.9.2 and Excel 2022.

### Total RNA extraction, quality inspection, cDNA library construction and sequencing

From each flower bud sample, 3 μg total RNA was extracted using Tiangen Polysaccharide and Polyphenol Kit (QIAGEN, Hilden, Germany). After passing the quality inspection, Illumina Novaseq 6000 (sequencing read length is PE150) was used for library construction and RNA-seq.

### Analysis of sequencing data

To ensure the quality and reliability of data analysis. Raw reads with an adaptor, or containing N (N indicates that base information cannot be determined), or with low quality (Qphred ≤20 base number accounts for more than 50% of the entire read length) were removed. Then cleaned high-quality reads were obtained. The reference genome sequence and annotation file of passion fruit were downloaded from the National Genome Data Center (NGDC) of China, project number PRJCA004251 (https://ngdc.cncb.ac.cn/gwh/Assembly/17982/show) accessed on 15 August 2022 (Xia et al., 2021). The clean reads were compared with the reference genome of passion fruit using HISAT2 software to obtain the information of reads on the reference genome (Mortazavi et al., 2008). The statistical power of this experimental design, calculated in RNAseq Power, was 0.91.

### Gene expression levels and differentially expressed gene enrichment analysis

Considering the influence of sequencing depth and gene length on the count of fragments, the FPKM value was used to estimate the gene expression level. The corrected read count value was used to screen the differentially expressed genes. Based on the negative binomial distribution, the DESeq2 R package (1.20.0) was used to screen differentially expressed genes (DEGs) with the criteria of Padj ≤0.05 (Padj is the corrected P) and |log2 (Fold Change)| ≥1. GO function and KEGG pathway enrichment analysis were performed on the differential gene sets using the cluster profiler (Yu et al., 2012).

### Validation of target gene using qRT-PCR

The complementary DNA (cDNA) was prepared by reverse transcription from cryopreserved passion fruit bud RNA using the cDNA Synthesis Kit (Thermo Fisher, Waltham, MA, USA). The expression of *EF1* and *Ts* in each tissue is relatively stable, so *EF1* and *Ts* were used as positive controls (Zhao et al., 2022; Tu, Fan & Xu, 2022). OLIGO was used to design specific primers for differentially expressed genes of passion fruit related to high-temperature stress. The primer sequences were delivered to Sangon Bioengineering (Shanghai, China) for synthesis (Table 1). The qRT-PCR assay was performed in a 20 μl of system: 0.4 μl of upstream and downstream primers (10 μmol·L^−1^), 1.0 μl of cDNA template, 10.0 μl of 2×SG Green qPCR Mix, 0.4 μl of ROX, and 7.8 μl of ddH_2_O. Amplification procedure: pre-denaturation at 95 °C for 10 min; denaturation at 95 °C for 15 s; annealing at 55 °C for 15 s; renaturation at 60 °C for 30 s; extension at 72 °C for 45 s; and a total of 45 cycles. The melting curve acquisition program: heating to 95 °C for 15 s; dropping to 60 °C for 30 s, and heating to 95 °C for 15 s. The amplification process was conducted in StepOne™ Plus Real-Time PCR System (Applied Biosystems, Waltham, MA, USA). The expression levels of differential genes were calculated by the 2^−ΔΔCT^ method (Tatarowska et al., 2020). Data were analyzed using Excel 2022 and Origin 2021.

**Table 1:** Primer sequence of identified RNA-seq by qRT-PCR.

Gene IDs	Forward primer (5′→3′)	Reverse primer (5′→3′)	Size (bp)
EF1 (Reference gene)	GGCTGAGCGTGAACGTGGTA	CGGCACAATCAGCCTGGGAA	146
Ts (Reference gene)	CAGTTGGTGCAGCCCATTGC	GGCCGTAACCTCGCGAATGA	93
maker-LG03-augustus-gene-1542.27	CGAGGATGGCGATTTGAAGC	CCACTGGTCCACTACAGCTC	127
snap_masked-LG01-processed-gene-802.5	TGCAGTTACCACAGTCATGGG	CTTCAGGTTGACAGGGGACTG	155
maker-LG02-snap-gene-1732.52	GCTTCAATGGGTCCGGAAGAA	ACAGGTTGCATTCTGGAGGC	92
maker-LG02-snap-gene-1798.82	TGTGTCGTAAGGGAAAGCCC	ACATGGCAAGAACTCCGAGG	138
maker-LG06-snap-gene-236.26	AGGAAAGCTAGAGGGCCAGA	CCAGGACAAGCCGTAGATCC	209
maker-LG02-augustus-gene-1357.20	CGCAGAGCTGATGAGGGAAT	CAACAGAGTTGGTCAGGCGA	143
maker-LG03-augustus-gene-159.26	GCGAGTGGATATTCGAGCCA	CAGTGATTGGGAACCCCGAA	193
maker-LG01-snap-gene-1853.58	ACGGTTCCCTCGTTAGATGC	AACAGTGGAAACGTGGACGA	124
maker-LG05-augustus-gene-960.1	CGGAGCAGAAGACAAAAGGGA	CAAGGTCACAGTTTGGACGG	73
maker-LG07-snap-gene-448.1	GCACGGGTTGTACGGAATTG	GGAACTCTTTCCGTCCTCCG	136

**Note:**

Gene IDs, the naming rules of genes are obtained according to the comparison results of reference genomes. The gene number is consistent with the reference genome; bp indicates the number of DNA base pairs.

### Data availability

The transcriptome sequence and annotation information of passion fruit buds measured in this study have been uploaded to the National Gene Bank of China (CNGB, CNP0003205) and the National Center for Biological Information (NCBI, PRJNA877848) in the United States for relevant researchers to consult.

## Results

### Changes in physiological and biochemical indexes of passion fruit in high-temperature conditions

The passion fruit undergoes a series of physiological and biochemical changes under high-temperature stress. The net photosynthetic rate, stomatal conductance, intercellular CO_2_ concentration, and transpiration rate of the high-temperature group were significantly lower than those of the control group (Table 2). Under high-temperature conditions, the passion fruit leaves quickly lose water. When the water deficit is severe; the content of abscisic acid in the leaves increases rapidly, which causes the stomata to close; and the stomatal conductance and photosynthetic rate to decrease accordingly. After the stomata is closed, the carbon dioxide needed for photosynthesis of passion flower can only be obtained between cells, and the carbon dioxide between cells is not replenicated in time, so the concentration decreases accordingly. In the healthy physiological state, the maximum photochemical quantum efficiency value of PSII of passion fruit is 0.815. However, the lowest measured value under high-temperature stress was 0.545, indicating that photosynthesis is affected and the poor health status. POD activity, SOD activity, and MDA content are critical indicators to measure the plant stress response. Among them, the MDA content is one of the essential indicators for judging the degree of damage to the plant cell membrane. Long-term high-temperature stress leads to a significant increase in the MDA content in the leaves of passion fruits, and the POD and SOD activities show varying degrees of change.

**Table 2:** Physiological and biochemical indexes of passionflower under two experimental conditions.

Category	Pn (μmol CO_2_m^−2^s^−1^)	Gs (molH_2_Om^−2^s^−1^)	Ci (μmol CO_2_ mol^-1^)	Tr (mmolH_2_Om^−2^s^−1^)	F_v_/F_o_	F_v_/F_m_	POD (U/μg)	SOD (U/μg)	MDA (nmol/g)
HT_1_	5.041	0.051	218.262	1.269	2.539	0.584	998.62	37.994	19.355
HT_2_	2.139	0.025	243.179	0.657	2.755	0.612	1569.26	17.532	13.011
HT_3_	4.675	0.048	219.228	1.203	2.699	0.545	3851.82	36.435	12.366
CK_1_	14.929	0.330	307.844	7.016	3.781	0.797	285.32	23.908	10.645
CK_2_	19.431	0.295	271.063	6.598	4.242	0.815	570.64	35.929	8.925
CK_3_	16.424	0.305	293.871	6.742	3.643	0.772	2853.20	17.160	6.882

**Note:**

HT, Sample materials after high temperature stress; CK, Sample materials of normal temperature control group; Pn, net photosynthetic rate; Gs, stomatal conductance; Ci, intercellular CO_2_ concentration; Tr, transpiration rate; Fv/Fo, photochemical potential activity of photosystemII; Fv/Fm, photochemical efficiency of photosystemII; POD, peroxidase activity; SOD, superoxide dismutase activity; MDA, malondialdehyde.

### Transcriptome sequencing analysis

The flower bud samples of the high-temperature group (HT_1_, HT_2_, and HT_3_) and the control group (CK_1_, CK_2_, and CK_3_) were sequenced, and a total of 41.31 G clean data were obtained. Each sample produced an average of more than 6.89 GB of high-quality data, The overall average sequencing error rate was 0.025. The percentages of Q20 bases and Q30 bases in each sample were greater than 97.85% and 93.84%, respectively, and the GC content ranged from 44.92% to 45.55%. The mapping rates of sequenced genes ranged from 77.71% to 86.10%, and an average of 74.49% of the reads could be aligned to the unique position in the genome (Table 3). The overall quality of the six samples met the requirements for subsequent analysis.

**Table 3:** Statistical results of transcriptome sequencing.

Sample	Clean reads	Clean bases	Error rate	Q20	Q30	GC pct	Total map	Unique map
HT_1_	46,977,170	7.05 G	0.02	98.08	94.34	45.55	85.37	75.22
HT_2_	45,134,840	6.77 G	0.03	97.92	93.97	45.31	80.58	71.62
HT_3_	44,519,812	6.68 G	0.03	97.88	93.91	45.25	82.96	75.10
CK_1_	44,885,248	6.73 G	0.03	97.85	93.84	45.5	77.71	69.15
CK_2_	46,352,310	6.95 G	0.02	98.1	94.39	44.96	86.10	78.64
CK_3_	47,502,264	7.13 G	0.02	98.04	94.24	44.92	84.32	77.19
Average	45,895,274	6.89 G	0.025	97.98	94.11	45.25	82.84	74.49
Total	275,371,644	41.31 G	0.15	587.87	564.69	271.49	497.04	446.92

**Note:**

HT, sample materials after high temperature stress; CK, sample materials of normal temperature control group; Clean reads, reads after filtering the original data; Clean bases, number of bases after filtering the original data; Error rate, overall sequencing error rate of data; Q20, percentage of bases with Phred value greater than 20 in total bases; Q30, percentage of bases with Phred value greater than 30 in total bases; GC pct, percentage of G and C in four bases in clean reads; Total map, comparison the number and percentage of reads on the genome; Unique map, the number and percentage of reads compared to the unique position of the reference genome.

### Screening of differentially expressed genes

**Figure 2:**
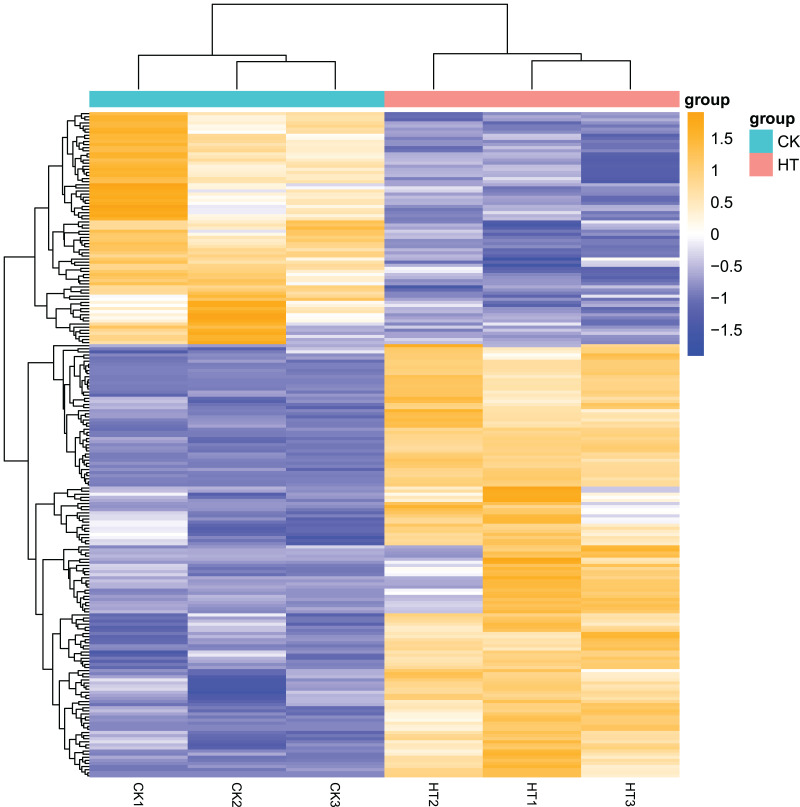
Heatmap of cluster analysis of DEGs from HT and CK transcriptomes. Cluster analysis was carried out on the differential gene set of *Passiflora edulis*, and the genes with similar expression patterns were clustered together (Annex S1). The abscissa of the figure is the sample name of passion fruit, and the ordinate is the normalized value of the differential gene FPKM. The closer the color is to orange, the higher the expression level, and the closer the color is to blue, the lower the expression level.

**Figure 3:**
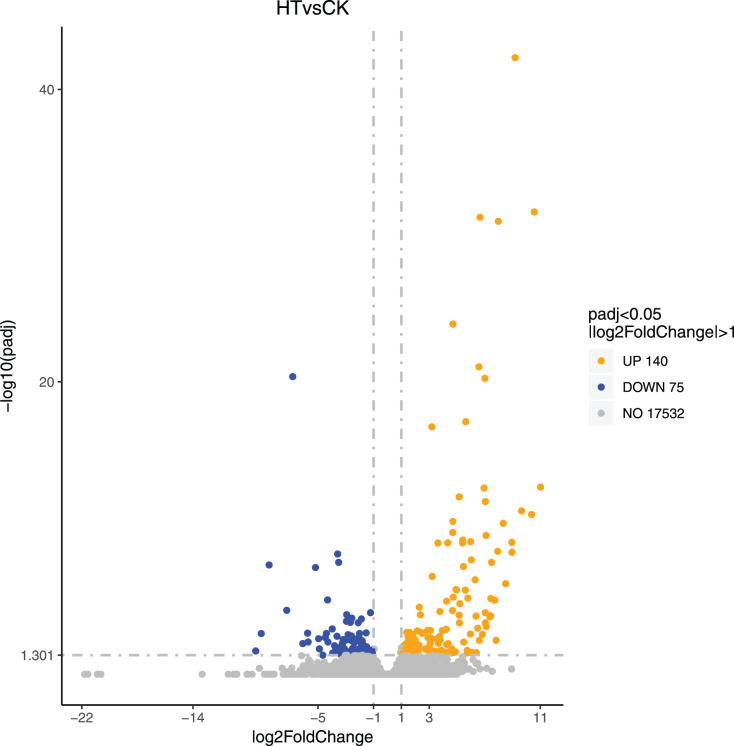
Volcano map of differentially expressed genes. Each dot represents a detected gene, and the abscissa is log2 (fold change). The more the dot deviates from the center, the greater the difference multiple. The ordinate is −log_10_ (*P*-value), and the closer the point is to the top of the graph, the more significant the difference is. Orange indicates that the corresponding gene expression is up-regulated, blue indicates that the corresponding gene expression is down-regulated, and grey indicates that there is no difference (Annex S2).

We performed differential expression analysis based on the sequencing results to find the gene response to high-temperature stress in passion fruit. Hierarchical clustering was used to perform bidirectional cluster analysis based on the FPKM values of the genes (Fig. 2). Statistical analysis was performed on the expression of the differentially expressed genes between the high-temperature group and the control group, and a total of 215 significantly differentially expressed genes were screened. Among them, 140 genes were up-regulated, and 75 genes were down-regulated. The up-regulated genes were about twice as many as the down-regulated genes (Fig. 3). Multiple genes showed differential expression patterns when passion fruit responded to high-temperature stress, indicating that the response to high-temperature stress was a complex process involved in multi-gene interaction.

### GO enrichment analysis of DEGs

**Figure 4:**
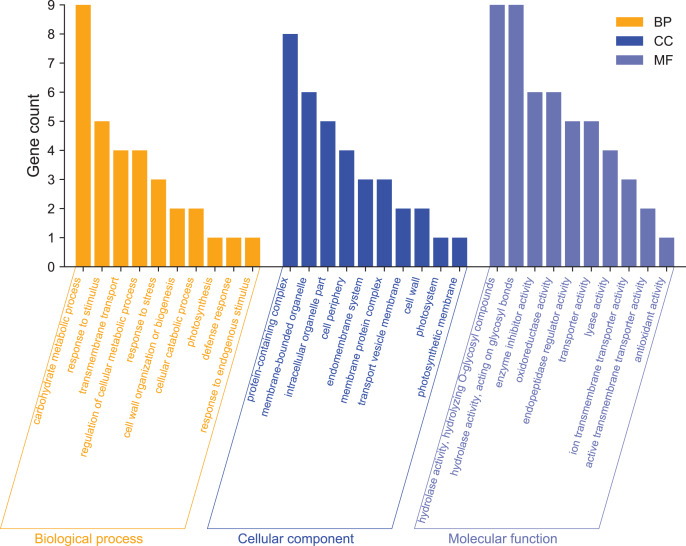
GO functional classification analysis of differentially expressed genes. The x-axis indicates the secondary classification terms of GO; the y-axis indicates the number of unigene in this secondary classification out of the total annotated unigenes.

GO enrichment analysis was used mainly to predict the function of differentially expressed genes. To further analyze the functions of differentially expressed genes, we performed GO function annotation analysis of 215 DEGs (Annex S3). We found that 195 DEGs were annotated into 331 categories, of which 134 were categories belonging to the biological process, 71 categories belonging to cellular components, and 126 categories belonging to molecular functions. Among them, 131 DEGs were up-regulated, and 64 DEGs were down-regulated (Annex S4 and S5). The GO term significantly enriched in the GO data of DEGs was Filter and summarize (Fig. 4, Annex S6). In terms of biological processes, in addition to carbohydrate metabolism (9, 13.04%, GO:0005975), which was the most significant enrichment, there were also stimulation responses (5, 7.24%, GO:0050896), transmembrane transport (4, 5.80%, GO:0055085), cellular metabolic process regulation (4, 5.80%, GO:0031323), stress-response regulation (3, 4.35%, GO:0006950), and other stress-related GO term factors. This indicates that the high-temperature has affected the normal life activities of passion fruit. In terms of cellular components, DEGs are mainly enriched in protein complexes (8, 33.33%, GO:0032991), membrane-bound organelles (6, 25%, GO:0043227), intracellular organelles (5, 20.83%, GO:0044446), and other GO terms. In terms of molecular functions, DEGs are mainly involved in hydrolase activity (9, 8.82%, GO:0004553), enzymatic activity regulation (6, 5.88%, GO:0030234), and oxidoreductase activity (6, 5.88%, GO:0016705). These results suggest that high-temperature stress impairs the metabolic reaction rate and normal physiological activities in cells. The cell can adapt to the changes in external conditions by regulating enzyme activity and maintaining its life activity.

### KEGG classification and pathway enrichment analysis of DEGs

**Figure 5:**
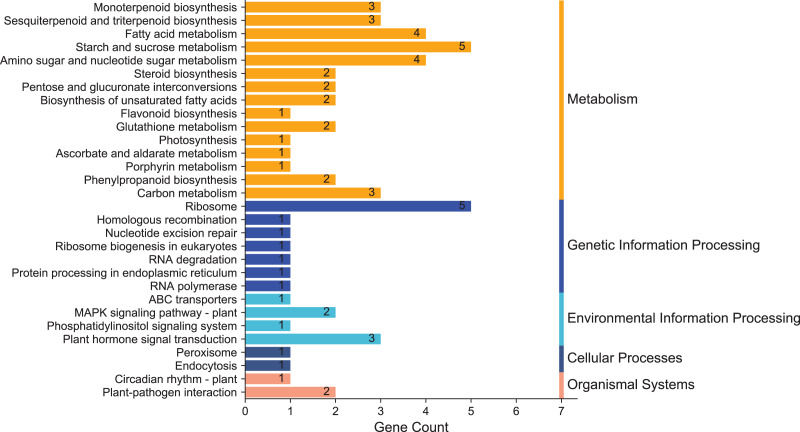
KEGG enrichment analysis of annotated DEGs. The x-axis indicates the number of unigenes in the pathway; the y-axis indicates KEGG pathways.

**Figure 6:**
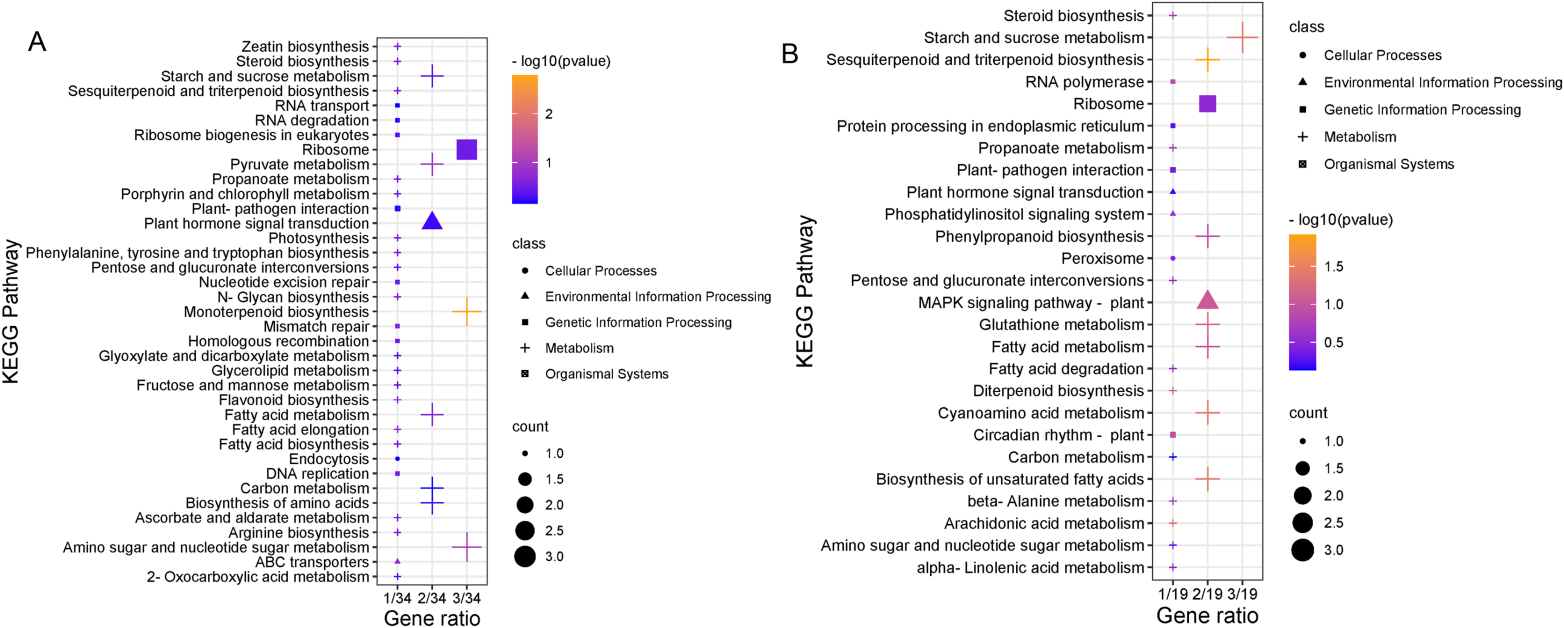
KEGG pathway enrichment analysis. (A) Results of up-regulating DEG pathway enrichment. (B) Enrichment results of DEG pathways down-regulated by expression level. The size of the shape indicates the number of genes in this pathway, and the color of the shape corresponds to different −log_10_ (*P* value) ranges.

To clarify the biological metabolic pathways of passion fruit in response to high-temperature stress, we performed a metabolic pathway regulatory network enrichment analysis of DEGs between the high-temperature group and the control group. A total of 53 DEGs were annotated to 52 KEGG metabolic pathways, including metabolism (34), genetic information processing (10), environmental information processing (four), cellular process (two), and biological system (two). Among them, 34 DEGs were up-regulated and 19 DEGs were down-regulated (Annex 7). The important metabolic pathways with the highest degree of DEGs enrichment were ranked (Fig. 5, Annex 8). The five metabolic pathways with a large number of DEGs annotations are starch and sucrose metabolism (five, 9.43%, ID: 00500), ribosome (five, 9.43%, ID: 03010), fatty acid metabolism (four, 7.55%, ID: 01212), amino sugar and nucleotide sugar metabolism (four, 7.55%, ID: 00520), and plant hormone signal transduction (three, 5.66%, ID: 04075). Based on the *P* value, we took a further metabolic pathway enrichment analysis of the up-regulated and down-regulated DEGs. It was found that the up-regulated DEGs in passion fruit buds were mainly related to monoterpenoid biosynthesis, amino sugar and nucleotide sugar metabolism, pyruvate metabolism, plant hormone signal transduction, porphyrin and chlorophyll metabolism, and photosynthesis under high-temperature conditions (Fig. 6A, Annex S9). DEGs with down-regulated expression were mainly enriched in sesquiterpene and triterpenoid biosynthesis, starch and sucrose metabolism, glutathione metabolic pathways, amino acid metabolism, unsaturated fatty acid biosynthesis, and MAPK signaling (Fig. 6B, Annex S10). Among them, plant hormone signal transduction, starch and sucrose metabolism pathways, porphyrin and chlorophyll metabolism, and photosynthesis pathways all play essential biological functions in response to a variety of plant stresses, which suggests that these metabolic pathways may be involved in responses to high-temperature stress.

### Candidate genes related to high temperature stress

Through the comparison of differentially expressed genes between the high-temperature group and the control group, followed by Gene Ontology (GO) and Kyoto Encyclopedia of Genes and Genomes (KEGG) enrichment analysis, and combined with relevant literature, we screened out 28 candidate genes that may be involved in high-temperature stress in passion fruit. These genes are involved in photosynthesis, phytohormone, glutathione, protein kinase, heat shock proteins, nighttime lighting and clock regulation, and thioredoxin (Table 4) (Wang, 2021; Hao, 2021; Chen et al., 2022).

**Table 4:** Differential expression of 28 candidate genes in passionflower under high-temperature stress.

Gene IDs	Gene name	Gene expression (FPKM)		log_2_ (Fold change)
		HT	CK	
Photosynthesis				
maker-LG06-snap-gene-236.26	PesCAO1	41.52	14.22	1.55
augustus_masked-LG06-processed-gene-154.56	PesCAO2	28.86	0.10	8.33
maker-LG03-snap-gene-1549.4	PesCAO3	16.38	0.63	4.70
maker-LG01-snap-gene-1050.8	PesCAO4	10.76	0.43	4.70
maker-LG02-augustus-gene-1357.20	PesCAO5	2.60	293.76	−6.82
snap_masked-LG06-processed-gene-149.9	PesCAO6	4.42	0.04	6.95
Plant hormone signal				
snap_masked-LG01-processed-gene-802.5	PesSAUR1	28.71	1.90	3.95
maker-LG02-snap-gene-1732.52	PesXTH1	15.06	1.66	3.19
maker-LG02-snap-gene-1798.82	PesPP1	3.03	17.07	−2.49
Glutathione				
maker-LG03-augustus-gene-1542.27	PesGST1	0.62	13.68	−4.47
maker-LG07-snap-gene-228.31	PesGST2	11.81	5.03	1.23
maker-LG06-snap-gene-57.0	PesGSH1	24.31	105.87	−2.12
Protein kinase				
maker-LG03-augustus-gene-159.26	PesPK1	40.64	7.35	2.47
maker-Contig20-augustus-gene-1.8	PesPK2	23.27	0	8.96
maker-Contig29-augustus-gene-1.94	PesPK3	7.75	3.03	1.35
maker-LG03-augustus-gene-151.65	PesPK4	4.31	0.54	3.04
maker-Contig5-snap-gene-4.19	PesPK5	1.84	0.14	3.75
augustus_masked-LG01-processed-gene-1682.46	PesPK6	1.47	0.18	3.06
maker-LG05-snap-gene-37.79	PesPK7	0.86	0.04	4.63
maker-LG02-snap-gene-1846.50	PesPK8	0.11	1.68	−3.94
Other				
maker-LG06-augustus-gene-1233.26	PesHSP1	0.96	0.06	4.14
maker-LG01-snap-gene-1801.51	PesHSP2	6.72	133.56	−4.31
maker-LG07-snap-gene-448.1	PesWRKY1	0.05	1.32	−4.66
maker-LG07-augustus-gene-269.0	PesWRKY2	0.34	0	6.08
maker-LG05-augustus-gene-960.1	PesLNK1	175.87	47.40	1.89
maker-LG01-augustus-gene-177.30	PesLNK2	96.70	41.56	1.22
maker-LG05-augustus-gene-227.12	PesLNK3	5.80	1.61	1.86
maker-LG01-snap-gene-1853.58	PesTRX1	105.92	35.19	1.59

**Note:**

Gene IDs, The naming rules of genes are obtained according to the comparison results of reference genomes. The gene number is consistent with the reference genome; Pes, Latin abbreviation of passion fruit; FPKM, Fragments Per Kilobase of exon model per Million mapped fragments; log2 (fold change), the ratio of expression between two samples (groups).

### Analysis of photosynthesis-related DEGs

Chlorophyll plays a role in photosynthesis and is essential for plant development processes and responses to environmental stimuli (Li et al., 2021). High-temperature stress leads to significant changes in photosynthesis-related energy metabolism and physiological processes. Compared to the control group, *PesCAO1*, *PesCAO2*, *PesCAO3*, and *PesCAO6* were up-regulated after high-temperature stress. It has been reported that *chlorophyllide a oxygenases (CAOs)* were involved in generating reactive oxygen species, which are signals and responses to abiotic stress. In this study, the expression level of chlorophyll a oxygenase *PesCAO1* was 41.52 in the high-temperature group, which was 2.92 times that of the control group (14.22). These results suggest that the passion fruit is more likely to maintain a high photosynthetic capacity under high-temperature stress.

### Analysis of plant hormone-related DEGs

High-temperature stress can cause changes in hormone metabolism and signal transduction in plant cells. Transcriptome sequencing analysis showed that the expression of auxin-responsive protein *PesSAUR1* and xyloglucan endotransglucosylase *PesXTH1* were up-regulated, while the expression of protein phosphatase *PesPP1* was down-regulated under high-temperature stress. These results indicate that different plant hormone response genes exhibit different expression patterns under high-temperature stress.

### Analysis of glutathione-related DEGs

Plant cells will produce excess reactive oxygen species under abiotic stress, resulting in oxidative stress. As a ubiquitous antioxidant in plants, glutathione can eliminate excess reactive oxygen species and keep cells in the homeostasis of redox reactions. The glutathione level is closely related to the tolerance of plants in the face of adversity. In this study, three differentially expressed genes were enriched in the glutathione metabolic pathway, including the glutathione peroxidase (*GSH*) gene and the glutathione S-transferases (*GST*) gene. These results suggest that passion fruit reduces the peroxidation of lipids in cells by regulating the expression of genes related to the glutathione metabolic pathway, thereby improving the tolerance to high-temperature stress.

### Analysis of protein kinase-related DEGs

Protein kinases mainly catalyze the phosphorylation of proteins, which is involved in the transmission of intracellular signals of plants under high-temperature stress. We found eight protein kinase-related genes that were up-regulated under high-temperature conditions. They may be involved in regulating phytohormone content, including auxin, abscisic acid, gibberellin, and cytokinin.

### Real-time fluorescence quantitative analysis

**Figure 7:**
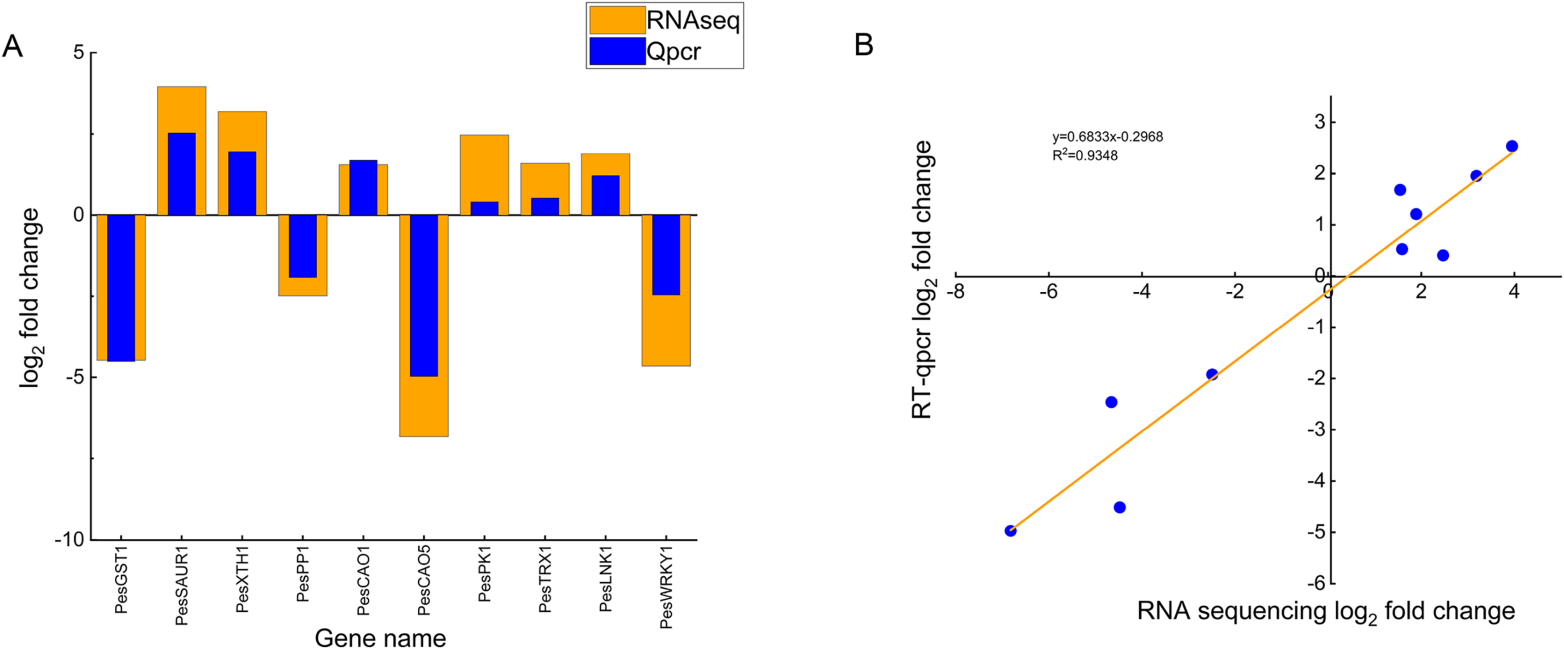
Comparison of real-time fluorescence quantitative expression and transcriptome sequencing of 10 DEGs. (A) The abscissa indicates the selected 10 differentially expressed genes related to high temperature stress of passion fruit, the ordinate indicates the differential expression multiples of RNAseq and Qpcr measured by genes, the column height indicates the differential expression multiples, and the smaller the height difference between orange and blue blocks indicates the more consistent the expression trend. (B) Each blue dot represents a differentially expressed gene, and the linear fitting of the differential expression multiples of RNAseq and Qpcr shows that the closer the R^2^ value is to 1, the higher the correlation.

To validate the reliability of the transcriptome sequencing data, we randomly selected ten DEGs (six up-regulated genes and four down-regulated genes) and verified them by qRT-PCR, including chloroplast genes (*PesCAO1*, *PesCAO5*), auxin-responsive protein gene *PesSAUR1*, Xyloglucan endotransglycosylase gene *PesXTH1*, protein phosphatase gene*PesPP1*, glutathione S-transferase gene *PesGST1*, protein kinase gene *PesPK1*, WRKY transcription factor gene *PesWRKY1*, night lighting and clock regulation gene *PesLNK1*, and thioredoxin gene *PesTRX1* (Fig. 7A). Correlation analysis showed that the changing trend and fold between real-time quantitative PCR and transcriptome data are generally consistent. The R^2^ is 0.9348, which confirms the reliability of the transcriptome sequencing results (Fig. 7B).

## Discussion

High temperature is one of the abiotic stress factors affecting crop production, which restricts the growth and development of crops. Studies have shown that high-temperature stress also changes the physiological and biochemical states, including decreased photosynthetic rate, blocked chlorophyll synthesis, and accumulated reactive oxygen species, which in turn cause a series of metabolic changes in cells. Once the plant cell membrane senses the high-temperature stress, cells initiate the transmission of signal molecules, causing changes in a series of life activities, such as enzyme activity, cell structure, gene expression, and regulation, to resist and ultimately alleviate the harm from the high temperature (Foyer & Noctor, 2005; Han et al., 2010). When the damage caused by the high-temperature stress exceeds the regulation ability of the organism, the plant begins to show the symptoms of heat damage in the form of appearance, such as the leaves turning yellow or curling, the fruit shrinking and losing water, and the branches withering and dying. Malondialdehyde content is an important indicator to evaluate the damage degree of the plant cell membrane system. In this study, the content of malondialdehyde in passion fruit was higher under a high-temperature stress, indicating that the cell membrane system was damaged under high-temperature stress of 38 °C. A previous study showed that the MDA content in kiwifruit under high-temperature stress significantly increased, and the results were consistent with ours (Dong, 2018). In this study, 215 differentially expressed genes related to regulating high-temperature stress were obtained at the transcriptional level based on RNA-seq technology. GO functional annotation analysis showed that the differentially expressed genes under high-temperature stress were mainly enriched in the biological process database of carbohydrate metabolism, stimulus-response, transmembrane transport, regulation of cellular metabolism, and stress response metabolism. In the GO cellular component database, differentially expressed genes are mainly enriched in protein complexes, membrane-bound organelles, and intracellular organelles. In the GO molecular function database, it is primarily enriched in hydrolase activity, enzyme activity regulation, and oxidoreductase activity. These results show that the response to high-temperature stress is a complex physiological process, and the resistance to damage caused by high-temperature stress requires the cooperation of multiple biological processes, cellular components, and molecular functions. In the KEGG enrichment analysis, 34 pathways were enriched, indicating that passion fruit enhances various metabolic activities to resist the damage caused by high temperatures. Another study reported that plant hormone signal transduction, glutathione metabolism, porphyrin and chlorophyll metabolism, the MAPK signaling pathway, and the photosynthesis pathway were involved in plant stress signal transduction (Jiang et al., 2021; Zhou et al., 2022). Our study also detected the high expression of differential genes in the metabolic pathways mentioned above, indicating that all of these signaling pathways respond to high-temperature stress in passion fruit. High temperature has a significant effect on the antioxidant enzyme system of capsicum, and the genes related to glutathione metabolic pathway can respond to high temperature stress quickly in a short time and participate in heat resistance regulation of capsicum (Wang et al., 2019). In this study, the results of KEGG metabolic pathway enrichment analysis showed that three differentially expressed genes were enriched in the glutathione metabolic pathway, and two genes were down-regulated under high temperature stress compared with the control group, suggesting that they play a role in the response of passion fruit to high temperature stress. Our study also found that the heat shock proteins *PesHSP1* and *PesHSP2* are involved in the response to high-temperature stress, which is consistent with the study using Lentinus edodes and strawberries (Xin, 2016; Zhang et al., 2022). Based on transcriptome sequencing, many studies have been conducted on the differential expression of genes under heat stress in crops. Although many differentially expressed genes related to high-temperature stress have been identified, most research sites are limited to crop leaves. Matsuda et al. (2020) pollinated passion fruit at different temperatures in the range of 28–42 °C and then recorded the fruit set rate and seed number to determine the critical high temperature that negatively affected the fruit set rate (Matsuda & Higuchi, 2020; Matsuda et al., 2020). The study found that the fruit set rate decreased significantly when the temperature was greater than 38 °C. They also found that the pollen tube of all pollinated flowers, which were incubated at 28–32 °C *in vitro*, was observed to reach the embryo sac within 24 h. When the culture temperature was higher than 34 °C, the shape of the pistils of all isolated flowers was disordered, and the pollen tube could not reach the embryo sac within 24 h. When pollinated at 40 °C, the pollen still germinated on the stigma but did not extend to the style within 24 h. Passion fruit buds often encounter high-temperatures during the bud differentiation period. High-temperature stress leads to a decrease in pollen vigor, which affects pollination and fruiting, resulting in a significant reduction in yield. Therefore, it is more targeted and innovative to choose the flower bud as the sequencing site to investigate the effects of high-temperature stress on the yield and quality of passion fruit.

## Conclusion

In this study, ‘Zhuangxiang Mibao’ was used as the test material to determine and analyze the leaves’ physiological indicators, which are related to the photosynthetic system of passion fruit under different temperature conditions, and transcriptome sequencing was performed on the flower buds. Under high-temperature stress, the stomatal conductance of passion fruit leaves decreased significantly, and MOD increased significantly. The results indicated the physiological and biochemical changes of passion fruit under high-temperature stress. Transcriptome sequencing analysis obtained some differentially expressed genes related to high-temperature stress. And then, we annotated the gene functions of the differentially expressed genes. There were 215 DEGs that specifically regulated between the high-temperature group and the control group, which were mainly related to hydrolytic enzyme activities and the metabolic pathways of carbohydrates, starch, and sucrose. All these genes are closely related to the high-temperature stress response. Based on the physiological changes of passion fruit leaves under high-temperature stress, candidate core gene groups, including *CAO*, *GSH*, *WRKY*, and *HSP*, were screened out. This may be involved in regulating the high-temperature stress response process of the passion fruit. Further, through transcriptome and fluorescence quantitative validation experiments, 10 differentially expressed genes were randomly selected for qRT-PCR validation. The dynamic analysis results were consistent with the results of the RNA-seq, indicating that the transcriptome data were reliable and the changes in gene expression and physiological indicators were consistent. Therefore, it is speculated that there may be a correlation between the changes in the physiological indexes and the expression of related genes of passion fruit under high-temperature stress. This study is a preliminary exploration of the response of passiflora to high-temperature stress. Our transcriptome data will be used as reference data for investigating passion fruit responses to high-temperature stress, such as molecular marker development, functional gene prediction, metabolic pathway exploration, and critical gene mining of high-temperature stress.

## Supplemental Information

10.7717/peerj.14839/supp-1 Supplemental Information 1 Heat map raw data of DEGs cluster analysis in HT *vs* CK transcriptome.

10.7717/peerj.14839/supp-2 Supplemental Information 2 Raw data of volcano map.

10.7717/peerj.14839/supp-3 Supplemental Information 3 Original data of 215 DEGs.

10.7717/peerj.14839/supp-4 Supplemental Information 4 131 up-regulated DEGs in GO enrichment analysis.

10.7717/peerj.14839/supp-5 Supplemental Information 5 64 down-regulated DEGs in GO enrichment analysis.

10.7717/peerj.14839/supp-6 Supplemental Information 6 The significantly enriched GO term and its corresponding gene number in the enrichment analysis.

10.7717/peerj.14839/supp-7 Supplemental Information 7 53 DEGs were annotated to 52 KEGG metabolic pathways.

10.7717/peerj.14839/supp-8 Supplemental Information 8 The important metabolic pathways with the highest degree of DEGs enrichment.

10.7717/peerj.14839/supp-9 Supplemental Information 9 Results of up-regulating DEG pathway enrichment.

10.7717/peerj.14839/supp-10 Supplemental Information 10 Enrichment results of DEG pathways down-regulated by expression level.

## Additional Information and Declarations

### Data Availability

The following information was supplied regarding data availability: The data is available at NCBI: PRJNA877848 (SRR21515660, SRR21515661, SRR21515662, SRR21515663, SRR21515664, SRR21515665). The data is also available at the CNGB Sequence Archive (CNSA) of China National GeneBank DataBase (CNGBdb): CNP0003205.

## References

[ref-1] Akhoundnejad Y, Dasgan YH, Karabiyik S (2020). Pollen quality, pollen production and yield of some tomato (Solanum lycopersicum) genotypes under high temperature stress in Eastern Mediterranean. Notulae Botanicae Horti Agrobotanici Cluj-Napoca.

[ref-2] Chen ST, Guo FQ (2013). Research progress of plant heat tolerance and heat shock signal transduction mechanism. Chinese Science. Life Sciences.

[ref-3] Chen XQ, Han J, Ren ZH (2021). Transcriptome analysis of cucumber in response to high temperature stress. Molecular Plant Breeding.

[ref-4] Chen Y, Yue LJ, Liu YH, Qin Y (2022). Effects of continuous high temperature treatment in vegetative growth period on transcriptome and biochemical indexes of maize leaves. Journal of Maize Sciences.

[ref-5] Dong X (2018). Study on the physiological effects of high temperature and strong light after continuous rainfall on kiwifruit.

[ref-6] Foyer CH, Noctor G (2005). Redox homeostasis and antioxidant signaling: a metabolic interface between stress perception and physiological responses. The Plant cell.

[ref-7] Guo LA, Mo RY, Tan J, Pan Y, Chen DX (2022). Transcriptome analysis of Pinellia ternata leaves under high temperature stress. Journal of Gansu Agricultural University.

[ref-8] Han MP, Gao YG, Wang CZ, Su FR, Wang YH, Zhang XX (2010). Study on the effect of high temperature stress on alfalfa and its adaptive mechanism. Genomics and Applied Biology.

[ref-9] Hao LH (2021). Transcriptome and function analysis of small heat shock protein gene in Paeonia suffruticosa under high temperature stress.

[ref-10] Huang CM, Luo HB, Huang YC, Wei YW, Yang L, Wu XJ, Ye LP, Cao HQ, Jiang SL (2021). Flower bud differentiation and physiological and metabolic effects of Passiflora edulis during fruit setting. Tropical Agricultural Science and Technology.

[ref-11] Huang Y, Shi XF, Lin L, Zhou SH, Han JY, Cao XJ, Zhang Y, Guo RR (2022). Subcellular localization, interaction protein screening and expression analysis of grape high temperature stress response gene BI1. Molecular Plant Breeding.

[ref-12] Jiang HY, Chrysanthemum Du, Shi LS, Bin J, Yue YZ (2021). Study on the molecular mechanism of plant response to high temperature stress. Molecular Plant Breeding.

[ref-13] Lan YP, Shi ZQ, Li LR (2021). Preliminary report of high-temperature experiment on the fruit setting rate of passion fruit. China Southern Fruit Tree.

[ref-14] Li Q, Zhou S, Liu W, Du H (2021). A chlorophyll a oxygenase 1 gene ZmCAO1 contributes to grain yield and waterlogging tolerance in maize. Journal of Experimental Botany.

[ref-15] Lu JN, Zhang D, Ding DD, Gao H, Han ZX, Liu X, Xiang L (2018). Study on the mechanism of high temperature promoting artemisinin biosynthesis in Artemisia annua. Chinese Journal of Traditional Chinese Medicine.

[ref-16] Matsuda H, Higuchi H (2020). Effect of temperatures on passion fruit flowering: a simulation model to estimate number of flowers. Tropical Agriculture and Development.

[ref-17] Matsuda H, Higuchi H, Okabe M, Ogata T (2020). Anatomical study for critical high temperature on the anthesis day to inhibit passion fruit set. Tropical Agriculture and Development.

[ref-18] Ma Y, Min L, Wang J, Li Y, Wu Y, Hu Q, Ding Y, Wang M, Liang Y, Gong Z, Xie S, Su X, Wang C, Zhao Y, Fang Q, Li Y, Chi H, Chen M, Khan AH, Lindsey K, Zhu L, Li X, Zhang X (2021). A combination of genome-wide and transcriptome-wide association studies reveals genetic elements leading to male sterility during high temperature stress in cotton. New Phytologist.

[ref-19] Mortazavi A, Williams BA, Mccue K, Schaeffer L, Wold B (2008). Mapping and quantifying mammalian transcriptomes by RNA-Seq. Nature Methods.

[ref-20] Qin ZC, Zhu JS, Feng Z, Chen YX (2017). Influence of adverse climate in Guilin on fruit setting of passion fruit and its countermeasures. Southern Horticulture.

[ref-21] Ren J, Wang Q, Zuo J, Jiang S (2021). Study of thermotolerant mechanism of Stropharia rugosoannulata under high temperature stress based on the transcriptome sequencing. Mycoscience.

[ref-22] Tatarowska B, Plich J, Milczarek D, Flis B (2020). Temperature-dependent resistance to potato virus M in potato (Solanum tuberosum). Plant Pathology.

[ref-23] Tian QL, Wu YY, Liu JY, Terry, Mou HF, Zhang YJ, Wei SL, Wei D (2021). Characteristics of Passiflora edulis yield and quality and its response to meteorological factors in Guangxi. Journal of Ecology.

[ref-24] Tian QL, Wu YY, Terry, Liu JY, Wei SL, Mou HF, Wei D, Huang YC, Xiong XL, Zhang YJ (2020). The flowering and fruit setting characteristics of Passiflora edulis ‘Tainong No.1’ and its relationship with meteorological factors. Journal of Fruit Science.

[ref-25] Tu ZH, Fan H, Xu M (2022). An internal reference gene of Passiflora edulis and its special primer and application. http://www.soopat.com/Patent/202010012116.

[ref-26] Wang J (2021). Evaluation of heat tolerance of pepper germplasm resources and study on its response mechanism to high temperature stress.

[ref-27] Wang HLZ, Hao JC, Lai M, Zhang YQ, Fu XF (2022). Research and analysis of passion fruit seedling industry in Qinzhou city, Guangxi. Journal of Tropical Agricultural Sciences.

[ref-28] Wang J, Lv J, Liu Z, Liu Y, Zou X (2019). Integration of transcriptomics and metabolomics for pepper (capsicum annuum L.) in response to heat stress. International Journal of Molecular Sciences.

[ref-29] Xia ZQ, Huang DM, Zhang SK, Wang WQ, Ma FN, Wu B, Xu Y, Xu BQ, Chen D, Zou ML, Xu HY, Zhou XC, Zhan RL, Song S (2021). Chromosome-scale genome assembly provides insights into the evolution and flavor synthesis of passion fruit (Passiflora edulis Sims). Horticultural Research.

[ref-30] Xin M (2016). Study on the changes of transcription expression and function of hydrophobic protein and heat shock protein in Lentinus edodes under high temperature stress.

[ref-31] Yu GC, Wang LG, Han YY, He QY (2012). ClusterProfiler: an R package for comparing biological themes among gene clusters. OMICS: A Journal of Integrative Biology.

[ref-32] Zhang SM, Qiu BY, Xu Q, Huang ZG, Luo TK (2022). Transcriptional analysis of the effect of exogenous calcium ions on strawberry seedlings under high temperature stress. Molecular Plant Breeding.

[ref-33] Zhao MQ, Fan H, Tu ZH, Cai GJ, Zhang LM, Li AD, Xu M (2022). Stable reference gene selection for quantitative real-time PCR normalization in passion fruit (Passiflora edulis Sims.). Molecular Biology Reports.

[ref-34] Zheng XQ, Wei FW (2018). Influence of unfavorable meteorological conditions on the production of golden passion fruit. Fujian Hot Crop Science and Technology.

[ref-35] Zhou N, Zheng Y, Lu JW, Hu Y, Tao WL, Lei KR, Pan XX (2022). Transcriptional analysis of radish seedlings under high temperature stress. Journal of South China Agriculture.

[ref-36] Zhu D, Li LH, Gao H (2020). Mining genes of peppermint seedlings in response to drought and high temperature stress based on transcriptome sequencing technology. Chemistry of Life.

